# When does embolism become too much? Crossing the point of no return and still surviving

**DOI:** 10.1093/plphys/kiag353

**Published:** 2026-06-10

**Authors:** Alice Gauthey, Alison Rose Gill

**Affiliations:** Assistant Features Editor, Plant Physiology, American Society of Plant Biologists; Birmingham Institute of Forest Research, University of Birmingham, Edgbaston B15 2TT, United Kingdom; Assistant Features Editor, Plant Physiology, American Society of Plant Biologists; Australian Research Council Centre of Excellence in Plants for Space, Adelaide University, Urrbrae 5074, SA, Australia

When does it become too much to handle? While this is an interesting philosophical question for humans, we could ask the same of trees. Trees face a limit beyond which recovery from stress is not possible, and for them, the issue is one of survival.

As climate change intensifies, drought stress is becoming both more severe and more frequent. When this stress exceeds critical thresholds, reduced soil water availability can drastically lower plants' water potential (Ψ_x_). This decline in Ψ_x_ can induce hydraulic failure: the continuous water column within xylem vessels breaks, allowing air bubbles, known as embolisms, to form and spread, progressively blocking water transport. When thresholds of embolism are exceeded, drought-induced hydraulic failure can lead to large-scale mortality (Choat et al 2012).

The Ψ_x_ threshold at which embolism develops and spreads is species-specific, and some species, particularly those adapted to arid environments, can resist embolism formation and therefore drought (Trugman et al 2021). The water potential at which 50% of hydraulic conductivity (PLC) is lost (Ψ_50_) is often used as an indicator of xylem vulnerability. However, there is still no clear consensus in the literature regarding the “point of no return” (ie, the level of embolism a tree can sustain while still remaining capable of recovery). Some studies suggest that mortality becomes inevitable after 60% to 90% of PLC within the xylem (Mantova et al 2021). Not only is this range remarkably broad, but the mechanisms underlying recovery remain debated. Some studies propose that, when transpiration ceases and water availability is restored, some plant species can refill embolized vessels (Rolland et al 2015). While this may occur in species capable of generating strong root pressure, recovery is more likely to rely on the growth of new xylem vessels that restore water transport (Gauthey et al 2022). The measure of recovery can also be assessed via morphological traits such as stem diameter or leaf production and also physiological traits such as the return of stomatal conductance (g_s_) values to predrought levels.

In a recent publication in *Plant Physiology*, Wagner et al (2026) investigated the effects of drought-induced embolism on drought recovery and associated physiological processes in carob seedlings (*Ceratonia siliqua*). This species is particularly interesting due to its capacity to resprout after drought, which is a key adaptation for prolonged drought survival (Zeppel et al 2015). The authors induced varying levels of drought stress by withholding water for different durations, followed by a 5-month recovery period after resuming watering. Using microtomography, they assessed the percentage loss of conductive area (similar to PLC) during both the drought and recovery phases. These measurements were integrated with physiological and morphological measurements, including Ψ_x_, g_s_, stem diameter, and leaf area. Wagner et al (2026) also estimated the lethal dose (LD_50_)—the Ψ_x_ or embolism amount leading to 50% mortality of the plants.

Carob seedlings were found to be very drought resistant, exhibiting an extremely negative Ψ_50_ of approximately −9.9 MPa. Despite their highly drought-resistant xylem, the LD_50_ still occurred at just below 40% PLC, with only a few individuals surviving at 70% (Fig. 1). As this LD_50_ is lower than what was previously reported in the literature (Urli et al 2013), it suggests that hydraulic resistance may not be the main limitation for drought survival. The authors therefore questioned the functional significance of investing in highly embolism-resistant xylem if such resistance is not consistently required for survival. They propose that carob's ability to resprout and its capacity for rapid postdrought recovery may be key to resolving this apparent mismatch. Yet, resprouting capacity declined with increasing embolism, as plants with greater hydraulic impairment showed a reduced ability to restore growth, even months after drought ended. Indeed, the authors showed that postdrought xylem growth was almost nonexistent in droughted plants compared with control plants, indicating that this species may not favor this costly mechanism, instead investing in more resistant xylem. This tradeoff between sensitivity to drought (ie, resistance) and postdrought recovery capacity (resilience) has been largely documented previously (Li et al 2020) but depends on factors such as growth rate tree size or environmental conditions. Successful resprouting in carob seedlings was strongly dependent on the plants' ability to supply water to the buds and developing leaves upon rewatering.

**Figure 1:**
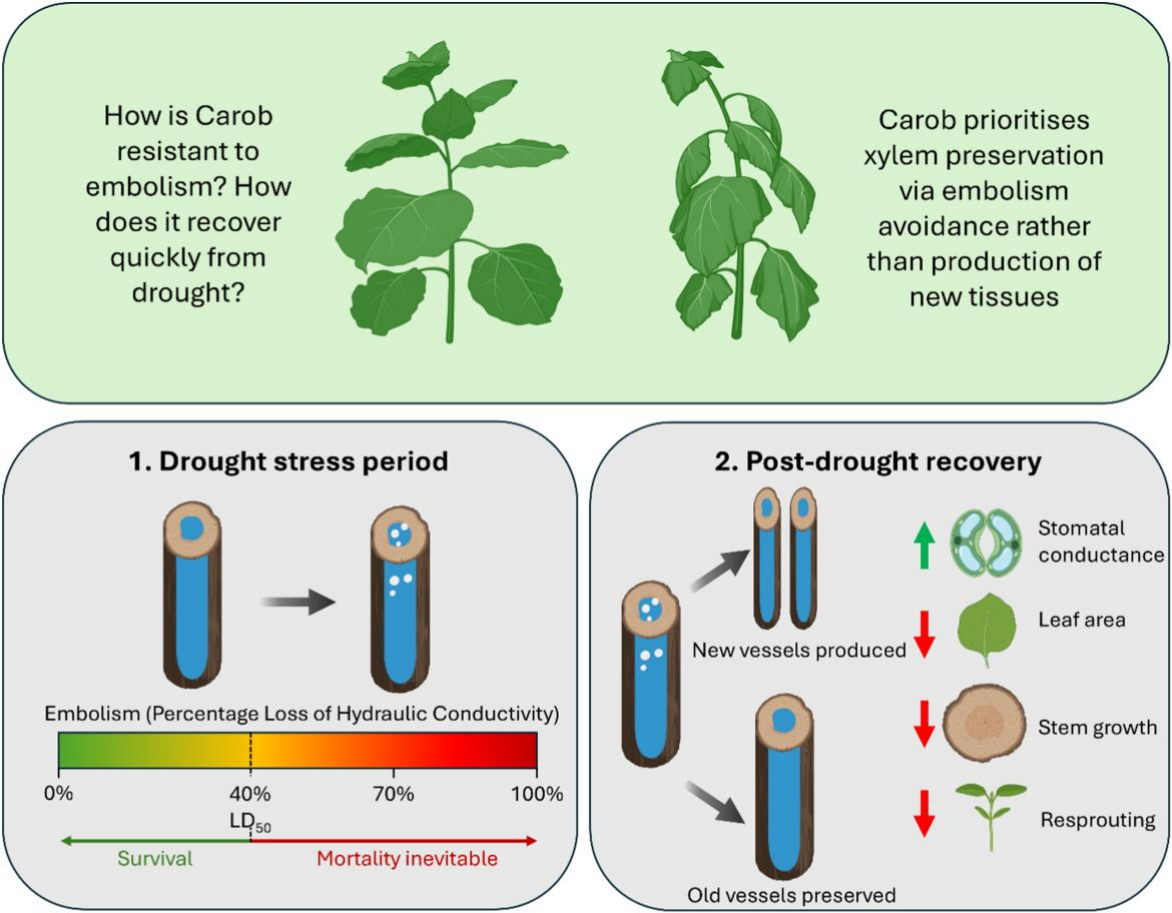
Schematic highlighting the responses to drought in carob seedlings, both during the stress period (1) and 5 months later (2). Embolism avoidance is critical for postdrought recovery in carob, but percentage loss of hydraulic conductivity (PLC) above 40% can be fatal. In (1), LD_50_ corresponds to the water potential at which 50% of plants are dead. The cylinders represent individual vessels filled with water (blue) and impacted by embolism (represented by white bubbles). In (2), the green arrow represents upregulation while red arrows represent downregulation of associated traits. Created in https://BioRender.com.

During recovery, the authors also noticed a lagged effect of the drought, whereby leaf area continued to decline during the month following rewatering. This reduction was more pronounced in seedlings exhibiting greater hydraulic impairment. Reduced leaf area in the most droughted plants persisted even after 5 months of recovery, despite restoration of the Ψ_x_ and g_s_ (Fig. 1). Similarly to leaf area, stem growth was strongly impacted by drought and did not fully recover within 5 months after rewatering. Drought legacies on tree growth or shoot recovery can indeed affect short-term recovery (Rehschuh et al 2020) and can last for several years following a drought event (Bréda et al 2006), especially if it occurs during the dry season and even when leaf-level gas exchange has been reestablished. Despite these results, Wagner et al (2026) found that embolism levels decreased by 13% to 38% during recovery. This reversal was likely limited, with carob prioritizing the preservation of xylem rather than producing new conductive tissues and with vessel refilling contributing only marginally to recovery.

In conclusion, the work by Wagner et al (2026) highlights the importance of understanding postdrought forest dynamics, a topic that is becoming increasingly relevant for forest regeneration as climates become hotter and drier. The authors emphasize the significance of the “point of no return,” which may be more variable than previously assumed. In addition, the embolism avoidance strategy observed in carob seedlings further illustrates the tradeoff between xylem resilience and resistance, as maintaining xylem integrity proved critical for drought recovery. Although this study focuses on seedlings, future work should characterize the ability of mature trees to recover from drought, particularly through resprouting and the preservation of xylem conductivity.

## Recent related articles in *Plant Physiology*

- Rehschuh et al (2020) reported drought legacy effects immediately after (2 days) or in the short-term following rewatering in *Pinus sylvestris* seedlings.
- Rolland et al (2015) showed that capillary forces were able to refill embolized vessels in 2 cushion plant species.

## Data Availability

Data can be found in the highlighted article and its online supplementary material.
